# Twisted Dual-Cycle Fiber Optic Bending Loss Characteristics for Strain Measurement

**DOI:** 10.3390/s18114009

**Published:** 2018-11-16

**Authors:** Sang-Jin Choi, Seong-Yong Jeong, Changhyun Lee, Kwon Gyu Park, Jae-Kyung Pan

**Affiliations:** 1 Petroleum & Marine Division, Korea Institute of Geoscience and Mineral Resources, 124 Gwahak-ro, Yuseong-gu, Daejeon 34132, Korea; sang-jin@kigam.re.kr (S.-J.C.); jetlee@kigam.re.kr (C.L.); 2 Department of Electrical Engineering and Smart Grid Research Center, Chonbuk National University, 567 Baekje-daero, Deokjin-gu, Jeonju 54896, Korea; jsy50541@jbnu.ac.kr

**Keywords:** fiber optic sensor, intensity-based fiber optic sensor, fiber reinforced plastics coupon strain, strain sensor, bending loss

## Abstract

The intensity-based fiber optic sensor (FOS) head using twisted dual-cycle bending loss is proposed and experimentally demonstrate. The bending loss characteristics depend on the steel wire radius, number, and distance. To determine the effects of these parameters, two samples in each of seven configuration cases of the proposed FOS head were bonded to fiber reinforced plastics coupons, and tensile and flexural strain tests were repeated five times for each coupon. The bending loss of the manufactured FOS heads was measured and converted to the tensile and flexural strain as a function of configuration cases. The measurement range, sensitivity, and average measurement errors of the tensile load and flexural strain were 4.5 kN and 1760 *με*, 0.70 to 3.99 dB/kN and 0.930 to 6.554 dB/mm, and 57.7 N, and 42.6 *με*, respectively. The sensing range of FOS head were 82 to 138 mm according to configuration cases. These results indicate that it is possible to measure load, tensile strain, and flexural strain using the proposed FOS head, and demonstrate that the sensitivities, the operating ranges, and the sensing range can be adjusted depending on the deformation characteristics of the measurement target.

## 1. Introduction

Over the last decade, aging structures have necessitated the development of techniques to ensure their safety, constituting a prominent aspect of structure monitoring. Assessing a structure by using a health-monitoring system can reduce the cost of maintenance and ensure safety [1]. Measurement factors for stability diagnosis in structural health-monitoring systems include cracks, surface degradation, acceleration, temperature, humidity, displacement, and strain. Excessive strain on a structure causes cracking and degradation of structural integrity, ultimately leading to collapse. Thus, measurement of the remaining fatigue lifetime of a structure is necessary to ensure its safety and can be achieved by conducting continuous strain monitoring [1].

Fiber optic sensor (FOS) technology has developed along with the growth of the optoelectronic and optical fiber telecommunication industry. An optical network can be used to obtain real-time measurement information. In addition, it can measure various types of measurement factors with arbitrary spatial distribution [2]. Generally, FOSs can be used to measure physical quantities, such as deformation, temperature, humidity, corrosion, and vibration. As structures have become larger, FOS-based schemes have received increasing attention in monitoring construction conditions.

Among fiber optic-based sensors, the intensity-based FOSs were the first to be developed due to their simplicity and potentially low cost. They continue to offer an attractive option in many sensing applications because they can measure a wide variety of parameters using inexpensive light sources and non-sophisticated detection schemes, while still benefiting from the intrinsic advantages of photonic sensors, i.e., their low weight, small size, and electromagnetic immunity [3,4]. An intensity-based FOS needs self-referencing characteristics to minimize the influences of long-term aging of source characteristics and to overcome short-term fluctuations. In addition, the sensor head needs to convert measurements such as strain, pressure, or force into the corresponding optical intensity change [4,5,6,7,8,9,10,11].

It is well known that radiation loss occurs when an optical fiber is bent [12,13,14,15,16]. Many polymer optical fibers (POFs) have utilized the attenuation of optical power caused by bending-induced mode conversion [16,17,18,19,20,21]. Lu et al. presented a POF displacement sensor that enhanced sensitivity using a bent and elongated grooved POF [16]. Kuang et al. presented a POF coupon subjected to dual cyclic bending that improved the sensitivity of the POF displacement sensor [17]. Wang et al. presented a novel means of transducing plantar pressure and shear stress using a sensor based on fiber optic bending loss with an FOS array [18]. Abe et al. reported a strain sensor that used a twisted optical fiber that measured the optical loss due to fiber curvature, in which the distributed strain along the sensor axis was converted into a distributed optical loss [19]. Zendehnam et al. investigated the characteristics of bending loss by studying the effect of bending radius, and the influence of the number of wrapping turns [20].

In this paper, an intensity-based FOS head, consisting of high carbon steel wires and a standard single-mode optical fiber, is proposed and applied to the measurement of fiber reinforced plastics (FRP) coupon tensile strain and three-point flexural strain. In Section 2, the FOS head design is presented, along with the twisted dual-cycle bending loss characteristics and a theoretical analysis. Section 3 presents the process for manufacturing the FRP coupon and the FOS head embedded on the coupon. Section 4 describes the experimental validation of the proposed FOS head, in which the bending loss characteristics were obtained via experimental measurements according to the distance between steel wires, wire radius, and the number of steel wires. The results of a tensile test and three-point bending test of the FRP coupon using the proposed FOS head are then provided, and can be used to manufacture FRP intensity-based sensing elements. Finally, we conclude the paper in Section 5.

## 2. Twisted Dual-Cycle Bending Loss Characteristics

The twisted dual-cycle bending structure for an intensity-based FOS head was designed using a standard single-mode optical fiber. The fiber optic bending loss characteristics of the designed twisted dual-cycle bending structure were then analyzed and experimentally demonstrated.

### 2.1. Twisted Dual-Cycle Bending Structure

**Figure 1:**
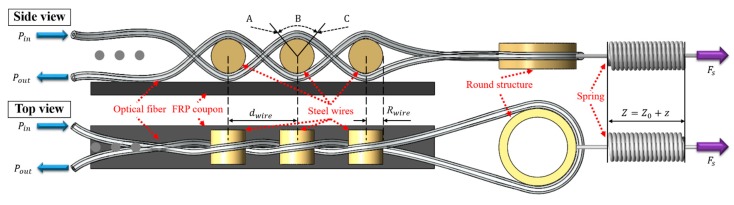
Schematic of the experimental setup for measuring the twisted dual-cycle bending loss characteristics ($d_{wire}$: distance between steel wires; $R_{wire}$: steel wire radius; Z: length of spring; $Z_{0}$: free length of spring; z: spring deflection length; $F_{s}$: spring tension load).

The proposed twisted dual-cycle bending structure for an FOS head that measures fiber optic bending loss is shown in Figure 1. The single-mode optical fiber is twisted around itself and is supported by high carbon steel wires mounted on an FRP coupon. The entire structure consists of the optical fiber, steel wires, a round structure, and a spring. The left side of the twisted optical fiber is fixed, and the right side consists of a turnaround structure where an external force ($F_{s}$) is applied to the optical fiber using the spring deflection length (z) shown in Figure 1. In the figure, the first cycle of the optical fiber bending route is from the input ($P_{in}$) to the round structure. The second cycle of the optical fiber bending route is from the round structure to the output ($P_{out}$), forming the dual-cycle bending loss structure.

The bending loss of an optical fiber includes both macro- and micro-bending losses. Macro-bending loss dominates when the curvature radius of the optical fiber is much larger than the diameter of the optical fiber [21]. The macro-bending loss increases exponentially when the curvature radius ($R_{b}$) is smaller than the critical radius ($R_{c}$) of the optical fiber [21]. Therefore, the critical radius plays a significant role in the design and application of optical fibers in sensing.

The critical radius ($R_{c}$) of a single mode optical fiber is given by [21]:

(1) $$R_{c}\approx 20\frac{\lambda }{{\left(\Delta \right)}^{3/2}}{\left(2.748-0.996\frac{\lambda }{\lambda_{cutoff}}\right)}^{-3}$$

where λ is the wavelength of the light, $\lambda_{cutoff}$ is the cutoff wavelength of an optical fiber, and Δ is the relative difference between the refractive index of the optical fiber core and cladding. A single mode optical fiber with a $\lambda_{c}$ of 1260 nm and a Δ of 0.00529 was used in our experiments. The calculated $R_{c}$ using Equation (1) is 22.85 mm. In the experimental setup shown Figure 1, the macro-bending loss dominates because the radii of the steel wires are smaller than the critical radius of the optical fiber.

**Figure 2:**
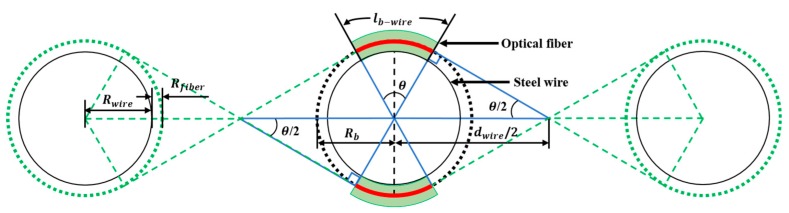
Schematic of the twisted dual-cycle bending length $l_{b-wire}$.

The total loss of a bent fiber includes the pure bending loss in the bent section and the transition loss caused by the mismatch in the propagation mode between the bent and straight sections. Figure 1 illustrates the macro-bending loss of the twisted optical fiber, which includes the pure bending loss in region B and the transition loss at positions A and C. When the inherent attenuation of the optical fiber is neglected, the optical power ratio of the output optical power, $P_{wire-out}$, to the input optical power, $P_{wire-in}$, for the passage of one steel wire in Figure 1 can be expressed as follows [21]:

(2) $$\frac{P_{wire-out}}{P_{wire-in}}=\left({\frac{P_{wire-out}}{P_{wire-in}}|}_{A}{\frac{P_{wire-out}}{P_{wire-in}}|}_{B}{\frac{P_{wire-out}}{P_{wire-in}}|}_{C}\right)$$

where losses A and C are the transition losses and loss B is the pure bending loss. Because a transition loss is much smaller than a pure bending loss for a certain wavelength, Equation (2) can be simplified as follows [21]:

(3) $${\frac{P_{wire-out}}{P_{wire-in}}=\frac{P_{wire-out}}{P_{wire-in}}|}_{B}=\exp\left(-2\alpha l_{b-wire}\right)$$

where α is the coefficient of the pure bending loss and $l_{b-wire}$ is the bending length of the optical fiber around one steel wire, as shown in Figure 2. The bending loss in the dB scale at the $l_{b-wire}$ section, $L_{single}$, using Equation (3) can be defined as follows [21]:

(4) $$L_{single}=10\log_{10}\left(\frac{P_{wire-in}}{P_{wire-out}}\right)=10\log_{10}\left[\frac{1}{\exp\left(-2\alpha l_{b-wire}\right)}\right]=4.342\left(2\alpha l_{b-wire}\right)$$

The bending loss coefficient, α, of a single mode optical fiber under weak guiding conditions is given by [22]:

(5) $$2\alpha =\frac{\sqrt{\pi }\kappa^{2}}{2\gamma^{3/2}V^{2}\sqrt{R_{b}}K_{+1}^{2}\left(\gamma a\right)}\exp\left(-\frac{2\gamma^{3}R_{b}}{3\beta^{2}}\right)$$

where κ is the normalized radial phase constant, γ is the normalized radial attenuation constant, V is the normalized frequency,$\;K_{+1}^{2}\left(\gamma a\right)$ is the modified Hankel function, a is the radius of the fiber core, and β is the axial propagation constant. These parameters are fixed values once the single-mode optical fiber and the wavelength of incident light are chosen. According to Equations (4) and (5), the bending loss of an optical fiber passing over one steel wire, $L_{single}$, is determined as follows [21]:

(6) $$L_{single}=4.342\left(\frac{A_{strc}}{\sqrt{R_{b}}}\exp\left(-B_{strc}R_{b}\right)\right)l_{b-wire}$$

where $A_{strc}=\sqrt{\pi }\kappa^{2}/2\gamma^{3/2}V^{2}K_{+1}^{2}\left(\gamma a\right)$ and $B_{strc}=2\gamma^{3}/3\beta^{2}$.

The bending length, $l_{b}$, of the twisted dual-cycle bending structure when the number of steel wires, $N_{wire}$, is can be expressed as follows:

(7) $$l_{b}=2l_{b-wire}N_{wire}=2R_{b}\theta N_{wire}=4R_{b}\sin^{-1}\left(\frac{2R_{b}}{d_{wire}}\right)N_{wire}$$

where $R_{b}$ is the sum of the steel wire radius, $R_{wire}$, and the optical fiber radius, $R_{fiber}$; $d_{wire}$ is the distance between two steel wires; and $\theta =2\sin^{-1}\left(\frac{2R_{b}}{d_{wire}}\right)$. Further, the bending loss, $L_{strc}$, given by the twisted dual-cycle bending structure using Equations (6) and (7) can be expressed as follows:

(8) $$L_{strc}=17.368N_{wire}A_{strc}\sqrt{R_{b}}\times \exp\left(-B_{strc}R_{b}\right)\sin^{-1}\left(\frac{2R_{b}}{d_{wire}}\right)$$

Equation (8) shows that the bending loss, $L_{strc}$, can be adjusted by changing $N_{wire}$, $R_{b}$, and/or $d_{wire}$.

### 2.2. Bending Loss Characteristics

The twisted dual-cycle bending loss characteristics were evaluated by measuring the optical power loss (dB) corresponding to the spring tension load ($F_{s}$) or the spring deflection length (z) using the experimental setup shown in Figure 1. In the experiments, we used a tunable laser (MG9638A, Anritsu, Atsugi-shi, Japan) with an input optical power of 0 dBm, wavelength of 1550 nm, and full width at half maximum (FWHM) of 0.2 nm; an optical power and energy meter (PM320E, Thorlabs, Newton, NJ, USA) with a fiber photodiode power sensor (S154C, Thorlabs); a single-mode optical fiber (SMF-28, Heesung Cable, Seoul, Korea) with an optical fiber radius, $R_{fiber},$ including polymer coating, of 0.1225 mm, an attenuation of 0.190 dB/km, and a polarization mode dispersion of 0.049 ps/$\sqrt{\mathrm{km}}$ at a wavelength of 1550 nm; and a tension spring (AWY8-60, Misumi, Tokyo, Japan) with an initial tension of 2.35 N and a reference spring constant of 0.2 N/mm.

**Figure 3:**
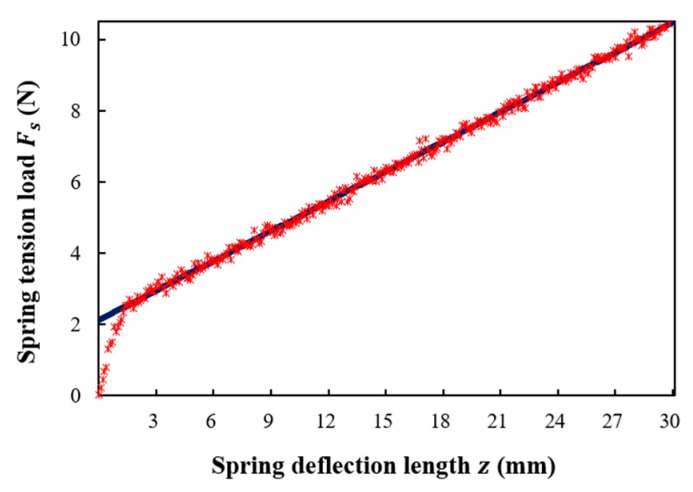
Measured spring tension load $F_{s}$ (N) versus spring deflection length z (mm).

Figure 3 shows the measured results of the spring tension load according to the spring deflection length in stepwise increments from 0.1 mm up to 30 mm via a universal testing machine (UTM) (INSTRON, 5982). The spring tension load increased linearly as the spring deflection length increased from 1.5 to 30 mm. The measured initial tension was 2.088 N, and the spring constant was 0.2781 N/mm. Therefore, the optical power loss (dB) versus spring deflection length (z) was determined using a spring tension load ($F_{s}$) within a linearly increasing range. An increase in the spring deflection length from 3 to 15 mm in 3 mm increments increased the spring tension load to 2.92, 3.76, 4.59, 5.43, and 6.26 N, respectively.

**Figure 4:**
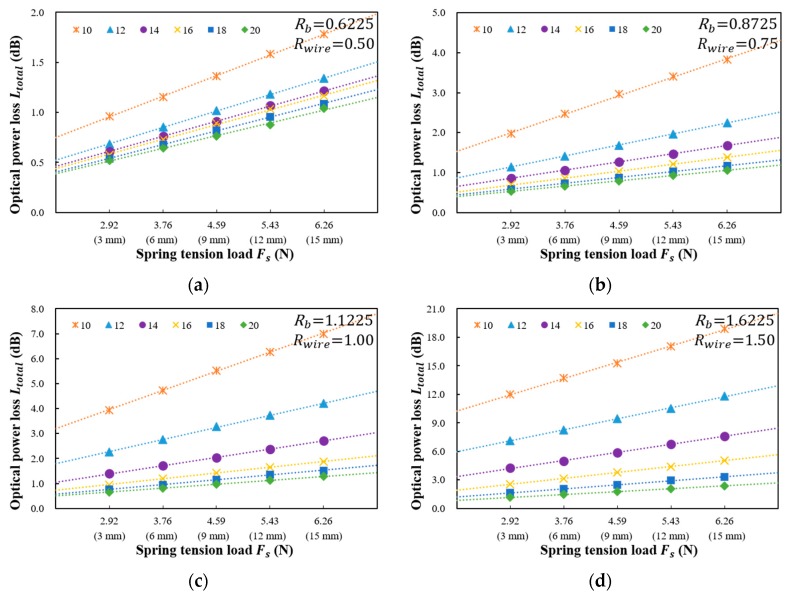

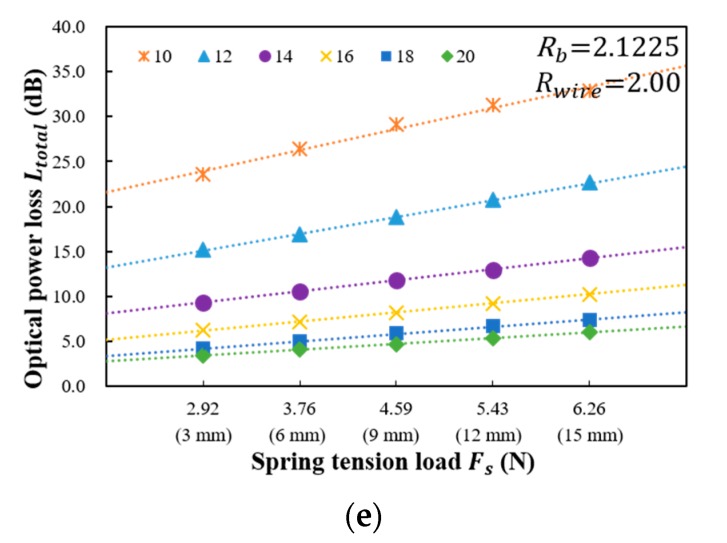
Measured optical power loss (dB) versus spring tension load $F_{s}$ (N) and spring deflection length z (mm) for various distances between the steel wires. The bending radii $R_{b}$ are (**a**) 0.6225 mm; (**b**) 0.8725 mm; (**c**) 1.1225 mm; (**d**) 1.6225 mm; (**e**) 2.1225 mm.

Figure 4 shows the results measured for the optical power loss versus the spring tension load and spring deflection length using five values of the bending radius ($R_{b}$ of 0.6225, 0.8725, 1.1225, 1.6225, and 2.1225 mm) and six distances between the steel wires ($d_{wire}$ of 10, 12, 14, 16, 18, and 20 mm) when $N_{wire}$ is 3. The relationship between the total optical power loss ($L_{total}$) and the spring tension load is linear. According to the Abe et al. the loss variation is linear when strain satisfies Δαε≪1 and a larger lateral rigidity of the primary coating (k), a smaller core radius (a) and a smaller refractive index deference (Δ) are necessary to achieve linearity for larger strains [19]. Because we used standard single mode optical fiber (SMF-28) that satisfies Δαε≪1, the optical power loss linearly increases with the increase of the tensile load.

The total optical power loss, $L_{total}$, for the proposed twisted dual-cycle bending structure can be expressed as:

(9) $$L_{total}=L_{strc}+F_{s}\times L_{force}$$

where $L_{force}$ is the optical power loss per unit Newton of the spring tension load. In Figure 4, the $L_{force}$ according to the sensor head structure can be obtained by using $L_{total}$ according to $F_{s}$. The value of $L_{strc}$ due to the twisted dual-cycle bending structure can be obtained by subtracting the loss due to $F_{s}$ from the measured value of $L_{total}$.

**Figure 5:**
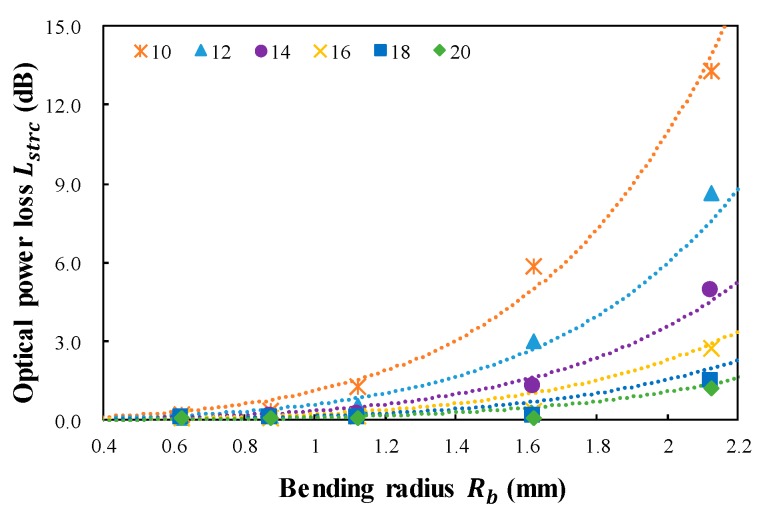
Calculated optical power loss ($L_{strc}$) versus bending radius ($R_{b}$) for six distances between steel wires ($d_{wire}$) of the twisted dual-cycle bending structure.

For the proposed twisted dual-cycle bending structure, the calculated optical power loss, $L_{strc}$, versus the bending radius, $R_{b}$, for six different distances between the steel wires, $d_{wire}$, is shown in Figure 5.

The optical power loss is increased by increasing $R_{b}$ and decreasing $d_{wire}$. When $d_{wire}$ is decreased, the bending length ($l_{b}$) in Equation (7) is increased. Ordinarily, increasing $R_{b}$ decreases the total optical loss because the optical power loss per unit length is decreased. However, our experiment demonstrated an increase in $L_{strc}$ when increasing $R_{b}$ due to the increase in $l_{b}$ in Equation (8). In this equation, $A_{strc}$ and $B_{strc}$ are the calculation results of the twisted dual-cycle bending loss for five different bending radii ($R_{b}$) and six distances between the steel wires ($d_{wire}$). Using the MATLAB exponential fitting model, $A_{strc}$ was determined to be 0.030 and $B_{strc}$ was determined to be −2.380. The exponential model has the largest multiple correlation coefficient with a value of 0.9816, and the root mean square deviation is 0.4064. Both $A_{strc}$ and $B_{strc}$ are constant values depending on the parameters of the twisted dual-cycle bending loss structure. Substituting these constant values for $A_{strc}$ and $B_{strc}$ in Equation (8) enables $L_{strc}$ to be predicted according to $d_{wire}$, $R_{wire}$, and $N_{wire}$.

**Figure 6:**
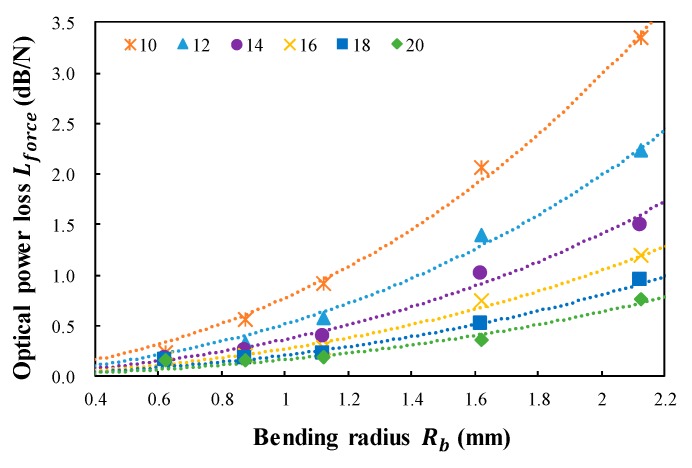
Calculated optical power loss per unit Newton ($L_{force}$) determined by the spring tension load ($F_{s}$) versus bending radius ($R_{b}$) for six distances between steel wires ($d_{wire}$) of the twisted dual-cycle bending structure.

Figure 6 plots the calculated results of the optical power loss per unit Newton ($L_{force}$) determined by the spring tension load ($F_{s}$) for five bending radii ($R_{b}$) and six distances between the steel wires ($d_{wire}$). The value of $L_{force}$ is also increased by increasing $R_{b}$ and decreasing$\;d_{wire}.$ Furthermore, the optical power loss per unit newton, $L_{force}$, can be expressed as in Equation (8) as follows:

(10) $$L_{force}=17.368N_{wire}A_{force}\sqrt{R_{b}}*\exp\left(-B_{force}R_{b}\right)\sin^{-1}\left(\frac{2R_{b}}{d_{wire}}\right)$$

Using the MATLAB exponential fitting model, $A_{force}$ was calculated to be 0.492 and $B_{force}$ was calculated to be −0.332. The exponential model has the largest multiple correlation coefficient with a value of 0.9932, and the root mean square deviation is 0.0642. Applying the constant value of $A_{force}$ and $B_{force}$ to Equation (10), $L_{force}$ can be predicted according to the $d_{wire}$, $R_{wire}$, and $N_{wire}$.

Figure 5 and Figure 6 indicate that the operating range of the twisted dual-cycle bend structure can be improved by decreasing $R_{wire}$ and/or increasing $d_{wire}$ while its sensitivity can be improved by decreasing $d_{wire}$ and/or increasing $R_{wire}$.

## 3. FOS Head Bonded to the FRP Composite Coupon

**Figure 7:**
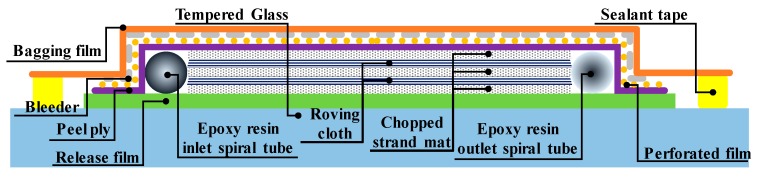
Schematic of the vacuum infusion bagging method.

Different FRP composites have different physical characteristics depending on the constitution of the fiberglass mat lay-up and manufacturing method [23]. Typically, the laminated structure of an FRP consists of chopped strand mat fabric and roving cloth fabric combined in an orderly manner. The FRP coupon used in this study was produced by the vacuum infusion bagging method using the infusion set up and glass fiber lay-up shown in Figure 7. In this lay-up, two roving cloth fabric sheets, constituting an anisotropic material, are located between three chopped strand mat fabric sheets that constitute an isotropic material. Thus, the composite material is composed of five sheets: two chopped strand mat fabric sheets with a thickness of 0.65 mm and a weight per unit area of 300 g/$m^{2}$, and the three roving cloth fabric sheets with a thickness of 0.5 mm and a weight per unit area of 570 g/$m^{2}$. The epoxy resin matrix material (RESOLTECH 1050, resoltech, Rousset, France) was mixed in a volume ratio of 7:3 with RESOLTECH 1056 hardener to manufacture the FRP composite coupon. The epoxy resin was injected using the vacuum infusion bagging method and cured at room temperature for 16 h.

In Figure 7, epoxy resin is injected through the inlet spiral tube located on the left side of the fiberglass mat, and a negative pressure of 95 kPa is applied by a vacuum pump connected to the outlet spiral tube located on the right side of the fiberglass mat. The vacuum pump removes air and unnecessary epoxy resin inside the compartment, which is otherwise sealed with bagging film and sealant tape. As a result, the prepared FRP composite material has a uniform thickness and surface quality, and the interlayer adherence, tensile strength, and impact strength are improved. Once produced, the manufactured FRP plate was cut into FRP coupons with a length of 200 mm and a width of 40 mm using an automatic cut-off machine (Brillant 220, ATM, Mammelzen, Germany) for testing using the UTM. The average thickness of the final FRP coupons was 2.2 mm.

**Figure 8:**
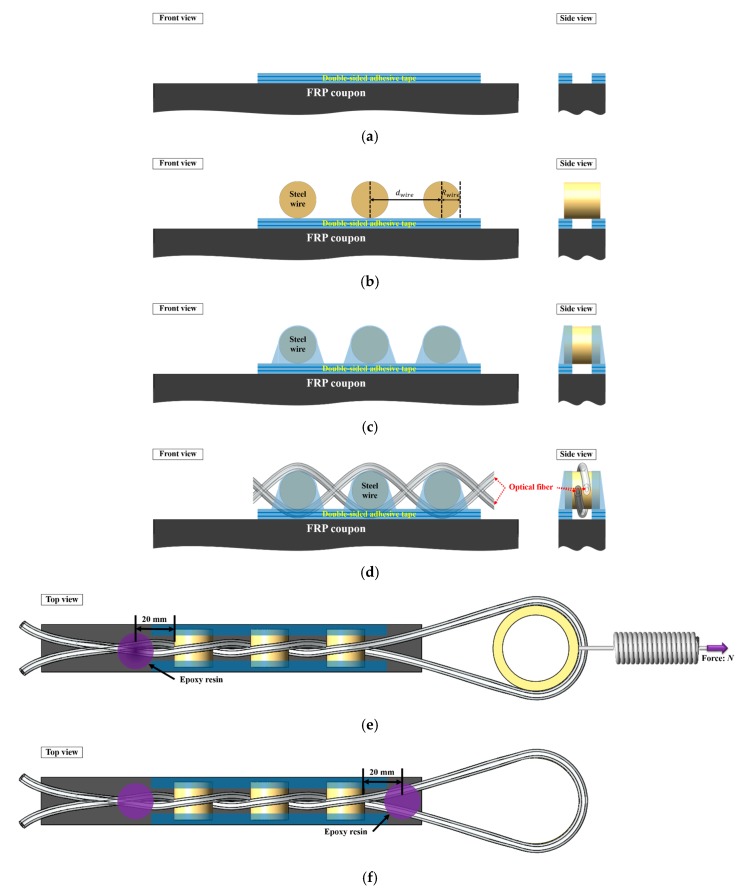
Intensity-based FOS head fabrication process.

The strain in the twisted dual-cycle bending structure was measured by attaching the proposed FOS head to the FRP coupon surface. The twisted dual-cycle bending structure shown in Figure 1 contains a standard single-mode optical fiber, the strands of which are twisted around each other as they travel between steel wires. This FOS head was installed on the surface of the FRP coupon using the procedure shown in Figure 8a–f and described as follows:

(a) Attach three sheets of double-sided tape (93015LE, 3M, Maplewood, MN, USA) in two strips to the surface of the FRP coupon. Each strip of double-sided tape fixes one end of the steel wire, leaving the middle part of the coupon without tape for the passage of the optical fiber.
(b) Place the steel wire on top of the double-sided tape under the conditions listed in Table 1. The sensor length depends on the number of steel wires and the distance between these wires.
(c) Fix both ends of the steel wire to the surface of the FRP coupon.
(d) Weave the single-mode optical fiber over and under the steel wires as they pass across the specimen.
(e) Fix the optical fiber to the FRP coupon surface with epoxy resin 20 mm from the left-most steel wire. After the epoxy resin is fully cured, connect the spring to the round structure for returning the optical fiber. Pull the spring to apply force to the optical fiber for setting the operating point of the sensor head that the optical power loss can be linearly operated according to the strain applied to the FRP coupons.
(f) Fix the optical fiber to the FRP coupon surface with epoxy resin 20 mm from the right-most steel wire. Remove the round structure and the spring after the epoxy resin has fully cured.

The force applied at the manufacturing process shown in Figure 8e is 3 N, which was derived from the analysis/investigation on the relation between power loss and tensile force shown in Figure 4. Actually, the magnitude of force applied during fabrication is important. If no force is applied at all or the force is too small, it is difficult to measure the optical power loss. On the other hand, if too much force is applied, insertion loss become larger.

Table 1 contains seven types of intensity-based FOS heads that were implemented using the above procedure and specifies the steel wire radius, number of steel wires, and distance between steel wires used in each head variant. As determined theoretically in Section 2, as the distance between steel wires and the number of steel wires increases, the sensing area increases, while as the steel wire radius and the number of steel wires increase, and the distance between steel wires decreases, the insertion loss increases.

## 4. Measurement of FRP Coupon Strain

The strain measurement experiment was designed and conducted using the configuration shown in Figure 1 with the proposed FOS heads in Table 1 based on the bending loss characteristics of the twisted dual-cycle configuration. The proposed FOS heads were used to collect measurements by bonding the twisted dual-cycle bending structure to the FRP coupon using epoxy resin, as described in Section 3, to conduct tensile strain and three-point bending tests. In these experiments, we used a tunable laser (MG9638A, Anritsu) with an input optical power of 0 dBm at 1550 nm and a full width at half maximum (FWHM) of 0.2 nm, an optical power and energy meter (PM320E, Thorlabs) with a fiber photodiode power sensor (S154C, Thorlabs), and a single mode optical fiber (SMF-28, Heesung Cable).

### 4.1. Tensile Strain Test

To determine the relationship between the FRP coupon tensile strain under the load $F_{t}$ versus the optical power loss of the FOS head, seven different tensile strength test cases were investigated using a UTM in load control mode. The optical power and FRP coupon tensile strain data from the UTM were recorded using a separate data acquisition system at a sampling rate of 2 Hz. The measurement started at an initial load of 50 N. The loading rate was 20 N/s and the maximum load was 4.5 kN with a measurement interval of 10 N. The measured load-strain relationships of the subject FRP coupons were linear: a tensile load of 4.5 kN is equivalent to an approximate tensile strain of 2000 με.

**Figure 9:**
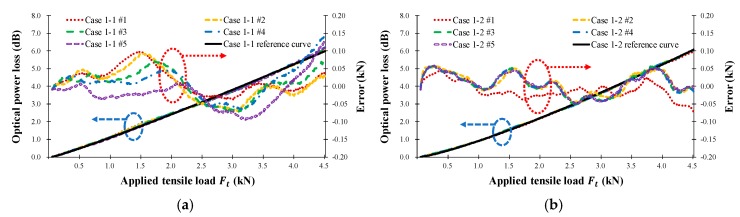

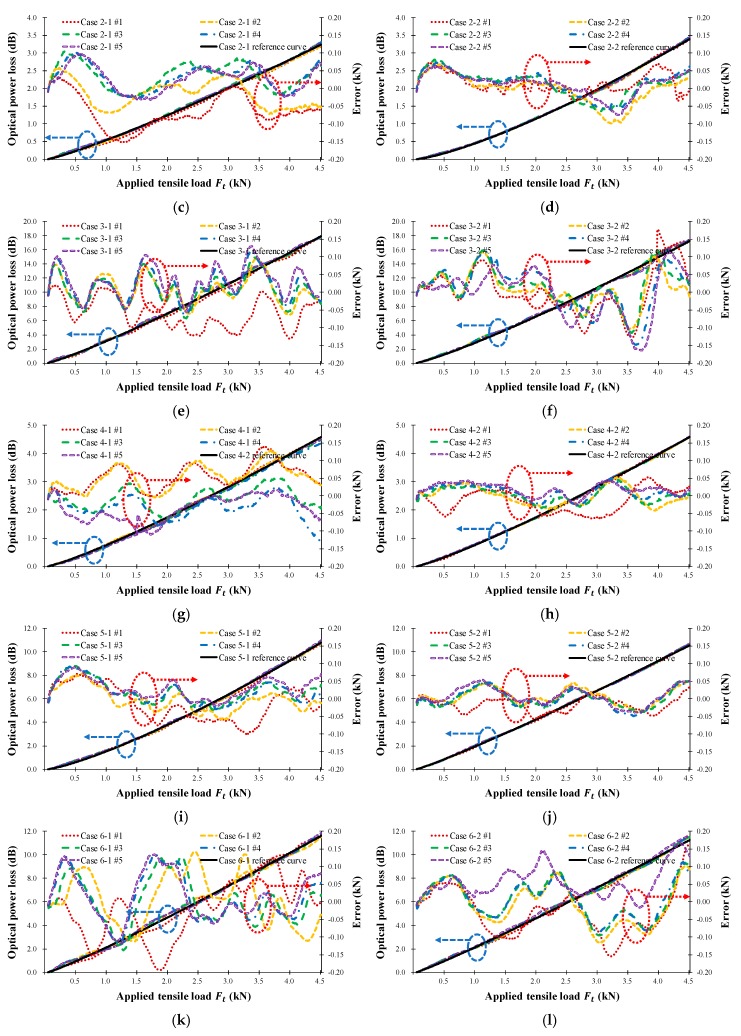

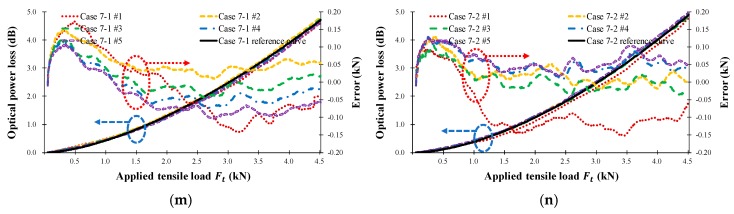
Measured FOS head optical power loss (dB) according to the applied tensile load $F_{t}$ (kN) for the seven types of FRP sensor head in Table 1. (**a**) Case 1-1; (**b**) Case 1-2; (**c**) Case 2-1; (**d**) Case 2-2; (**e**) Case 3-1; (**f**) Case 3-2; (**g**) Case 4-1; (**h**) Case 4-2; (**i**) Case 5-1; (**j**) Case 5-2; (**k**) Case 6-1; (**l**) Case 6-2; (**m**) Case 7-1; (**n**) Case 7-2.

The bending loss characteristics depend on the steel wire radius, numbers, and distance. To determine the effects of these parameters, two FRP coupons were tested for each of the seven cases and each coupon test was repeated five times. Table 1 provides the details of the twisted dual-cycle bending structure test cases for each of the coupons. Figure 9 shows the measured optical power loss (left) and error (right) of the FOS head versus the applied tensile load ($F_{t}$) for each of the seven cases. The black solid line is the calculated reference curve plotted using the MATLAB curve-fitting tool for one-term power series models ($s\times F_{t}{}^{l}$). Table 2 provides the results of the tensile tests, in which the parameters s and l represent the loss characteristics of the FOS head corresponding to the load or strain. These are correction variables for converting the optical power loss measured by the optical power meter into the measured load or strain applied to the FRP coupon upon which the sensor head is installed. When the s and l parameters are applied to the optical power loss occurring in the sensor, the applied tensile load, $F_{t}$, can be expressed as follows:

(11) $$F_{t}={\left(L_{tensile}/s\right)}^{1/l}\;\left[\mathrm{kN}\right]$$

where $L_{tensile}$ is the optical power loss of the FOS head according to the tensile load applied to the FRP coupon.

The tensile load calculated using Equation (11) was compared with that applied by the UTM and is indicated by the error axis in Figure 9. The average error of the load measured by the FOS heads is 57.7 N and the average error of the tensile strain is less than 26.1 *με*. Clearly, it is possible to measure the load and tensile strain by using the proposed intensity-based FOS head.

**Figure 10:**
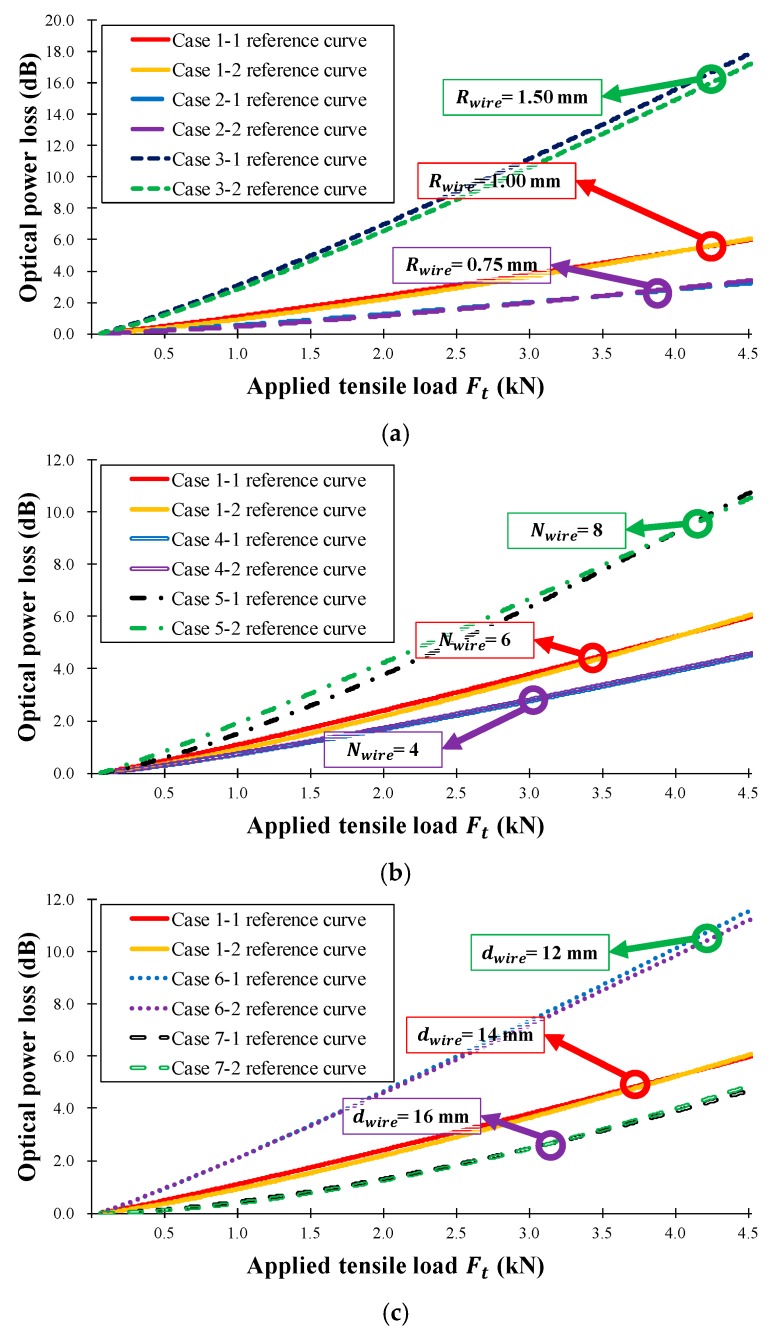
Measured optical power loss (dB) versus applied tensile load $F_{t}$ (kN) for the manufactured FOS heads in Table 1. (**a**) $R_{wire}$ = 0.75, 1.00, and 1.50 mm, $N_{wire}$ = 6, $d_{wire}$ = 14 mm;(**b**) $R_{wire}$ = 1.00 mm, $N_{wire}$ = 4, 6, and 8, $d_{wire}$ = 14 mm; (**c**) $R_{wire}$ = 1.00 mm, $N_{wire}$ = 6, $d_{wire}$ = 12, 14, and 16 mm.

Figure 10 shows the calculated reference curve for the seven test cases, while Table 2 presents the calculated results of the one-term power series models (s and l), regression analysis (R-square), and Root Mean Squared Error (RMSE). In Table 2, the R-square values were measured five times, producing a minimum R-square value of 0.997; therefore, the calculated reference curve can be considered a good representation of the characteristics of the seven sensor head cases. Table 2 also indicates that increasing the sensor head sensitivity increases the R-square, RMSE, and average error values. Figure 10a shows the optical power loss as a function of the wire radius, $R_{wire}$, in Equation (8): as the wire radius increases, the sensitivity of the sensor head increases, and the average error is not affected. Figure 10b displays the loss depending on the number of wires, $N_{wire}$, in Equation (8). As the number of wires increases, the sensitivity of the sensor head increases, and the average error is nearly constant. Additionally, as the number of wires increases, the sensor length increases, and thus the measurement range also increases. Figure 10c shows that the sensitivity of the sensor head decreases as the distance between wires, $d_{wire}$, in Equation (8) increases because of the bending length ($l_{b}$) in Equation (7), and because the measurement range is increased by increasing the sensor length. In summary, the sensitivity of the twisted dual-cycle bending structure can be adjusted by changing the radius of the steel wires, the number of steel wires, and the distance between these steel wires.

### 4.2. Three-Point Bending Test

**Figure 11:**
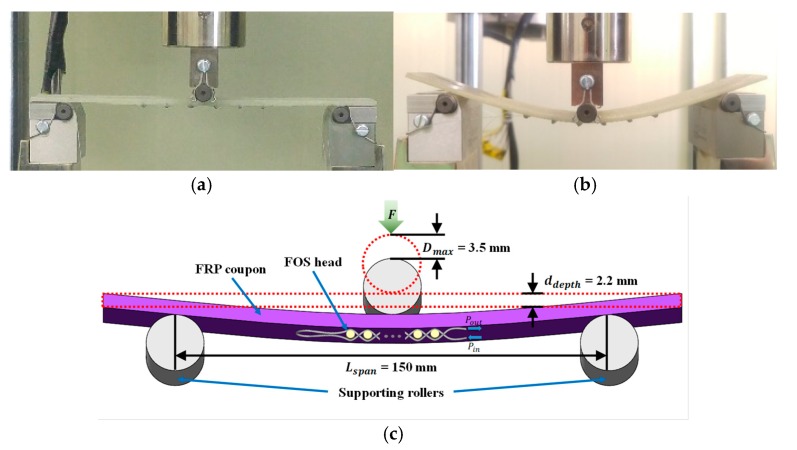
Three-point bending test setup with (**a**) unloaded image of the FOS head installed in the UTM; (**b**) loaded image of the FOS head installed in the UTM; (**c**) Schematic of the FOS head placement.

In the three-point bending test, strain was applied to an FRP coupon embedded with the proposed twisted dual-cycle bending structure to investigate the relationship between the optical power loss of the FOS head and the applied flexural strain. The bending loss characteristics depend on the steel wire radius, numbers, and distance. To determine the effects of these parameters, two FRP coupons were tested for each of the seven cases detailed in Table 1, in which the steel wire radius, number of steel wires, distance between steel wires, total embedment length, and insertion loss (dB) is varied. Figure 11 shows image of the FOS head installed in the UTM under unloaded and loaded condition and schematic of the three-point bending test setup where the FOS head adhered to the center of the FRP coupon as described in Figure 8. The FRP coupon was subjected to increasing cyclic loads following a triangularly shaped deflection pattern applied by the UTM. The diameter of the vertically moving loading roller and horizontally moving supporting rollers was 10 mm. The experiment measured the FRP coupon deflection, which we plotted it against the optical power loss measured by the proposed FOS head. The FRP coupon was loaded at a vertical displacement rate of 0.04 mm/s in measurement intervals of 0.02 mm until the deflection reached 3.5 mm from the initial 0.5 mm deflection.

The theoretical flexural strain in the outer surface of the FRP coupon, $\;\epsilon_{f}$, can be expressed as [24]:

(12) $$\epsilon_{f}=\frac{6Dd_{thickness}}{L_{span}^{2}}$$

where D is the deflection of the center of the FRP coupon under increasing cyclic loading following a triangularly shaped deflection from 0.5 mm to 3.5 mm, $d_{thickness}$ is the depth or thickness of the tested FRP coupon (≅2.2 mm), and $L_{span}$ is the support span (≅150 mm). From Equation (12), we can see that the flexural strain and deflection have a linear relationship. The change in$\;\epsilon_{f}$ between the maximum and initial deflection was 1760 με and the deflection sensitivity was 586.7 με/mm. The calculated flexural strain in the outer surface of the FRP coupon was then used as a reference value to verify the performance of the FOS head.

The correction parameters h and k are used to represent the loss characteristics of the FOS head corresponding to the flexural strain, allowing the optical power loss measured by the optical power meter to be converted into flexural strain. The experimental flexural strain can be obtained by applying the parameters h and k to the measured optical loss occurring in the sensor head as follows:

(13) $$\epsilon_{f}={\left(L_{flex}/h\right)}^{1/k}$$

where $L_{flex}$ is the optical power loss of the FOS head due to the flexural strain applied to the FRP coupon.

**Figure 12:**
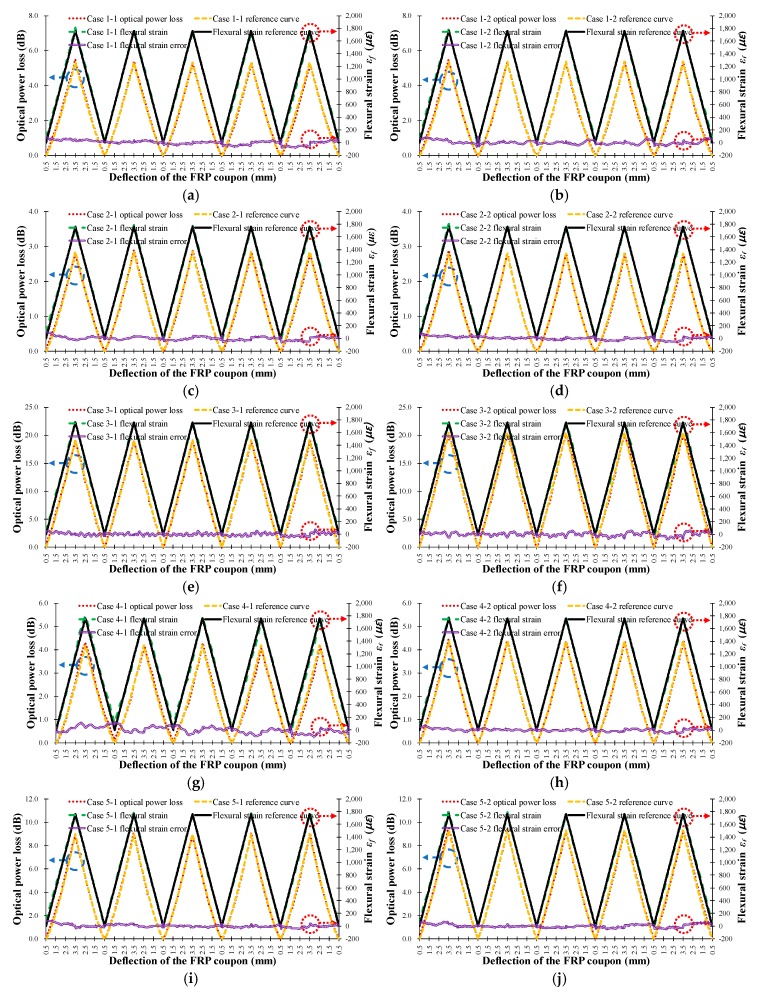

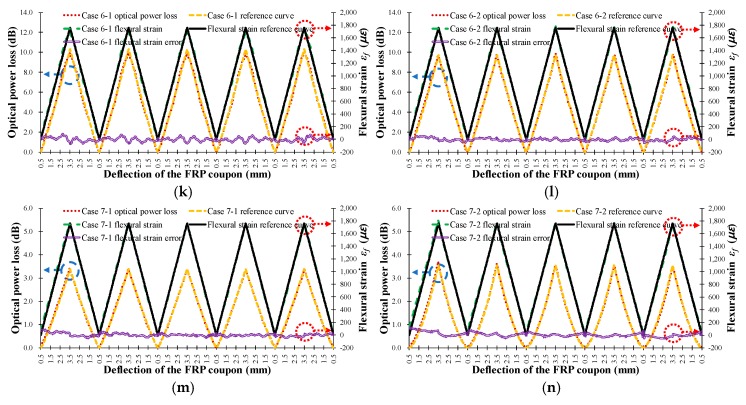
Measured FOS head optical power loss (**left**), measured flexural strain, and calculated flexural strain error (**right**) according to the deflection of the FRP coupon for the seven types of FRP sensor head in Table 1. (**a**) Case 1-1; (**b**) Case 1-2; (**c**) Case 2-1; (**d**) Case 2-2; (**e**) Case 3-1; (**f**) Case 3-2; (**g**) Case 4-1; (**h**) Case 4-2; (**i**) Case 5-1; (**j**) Case 5-2; (**k**) Case 6-1; (**l**) Case 6-2; (**m**) Case 7-1; (**n**) Case 7-2.

Figure 12 shows the measured optical power loss of the FOS head (left vertical axis, red dotted line) and theoretical $\epsilon_{f}$ in the outer surface (right vertical axis, black solid line) versus the deflection of the center of the FRP coupon for the seven cases in Table 1. The experimentally determined flexural strain values (right vertical axis, green dotted line) using Equation (13) and the theoretical flexural strain calculated using Equation (12) was compared and is indicated by the flexural strain error (right vertical axis, purple double solid line) in Figure 12, where it can be seen that the experimental flexural strain is nearly equivalent to the theoretical flexural strain, regardless of the characteristics of the sensor head. Therefore, it can be concluded that the optical loss occurring in each sensor head can be accurately converted into flexural strain by using Equation (13). The error in the flexural strain increases abruptly when the deformation of the FRP coupon changes direction, i.e., from increasing to decreasing deflection or from decreasing to increasing deflection, as indicated by the experimental error. The average error in the flexural strain measured by the FOS heads was less than 42.6 *με*. Thus, it is possible to accurately measure the flexural strain in FRP by using the proposed intensity-based FOS head.

**Figure 13:**
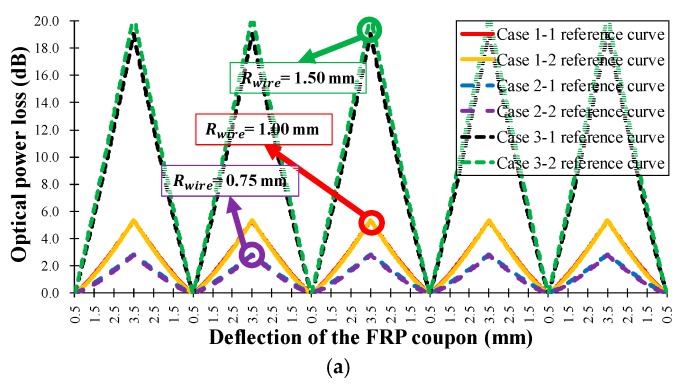

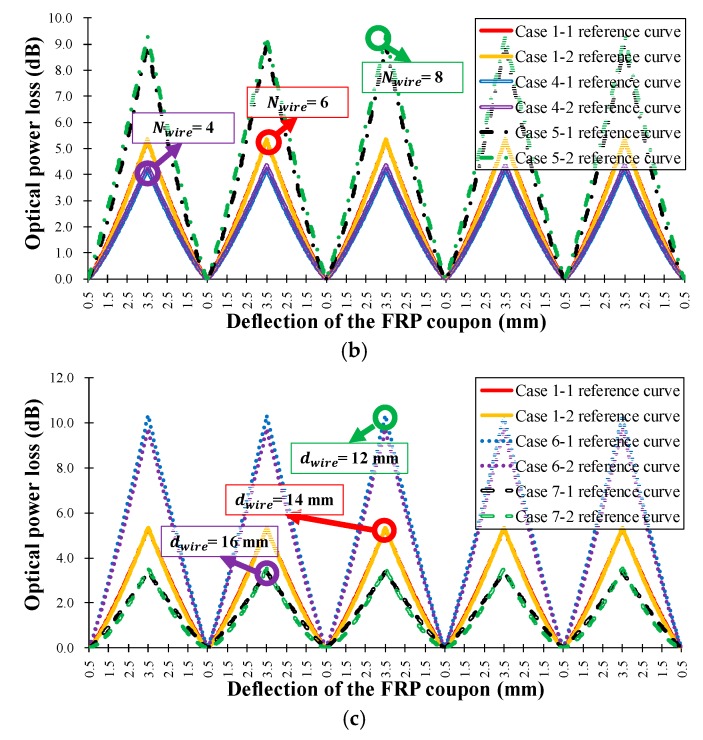
Measured FOS head optical power loss according to the three-point bending deflection of the FRP coupon of the seven types of FRP sensor head in Table 1. (**a**) $R_{wire}$ = 0.75, 1.00, and 1.50 mm, $N_{wire}$ = 6, $d_{wire}$ = 14 mm; (**b**) $R_{wire}$ = 1.00 mm, $N_{wire}$ = 4, 6, and 8, $d_{wire}$ = 14 mm; (**c**) $R_{wire}$ = 1.00 mm, $N_{wire}$ = 6, $d_{wire}$ = 12, 14, and 16 mm.

Figure 13 shows the optical power loss of the sensor head as a function of the steel wire radius, the number of steel wires, and the distance between steel wires. Figure 13a shows the optical power loss according to the wire radius, $R_{wire}$, in Equation (8). As the wire radius increases, the sensitivity of the sensor head increases because the angle of contact between the steel wire and the optical fiber has increased. The sensitivity of the FOS head can therefore be adjusted by varying the radius of the steel wires without changing the length of the sensor head. Both the tensile strain test and the flexural strain test displayed an increase in the average sensitivity when the steel wire radius was increased. Figure 13b shows the dependence of optical power loss on the number of wires, $N_{wire}$, in Equation (8). As the number of wires increases, the sensing area and the sensitivity increase because the total bending length ($l_{b}$) of the optical fiber increases. Figure 13c shows the decrease in sensitivity of the sensor head as the wire interval, $d_{wire}$, in Equation (8) is increased and the angle of contact between the steel wire and the optical fiber accordingly decreases.

The results in Figure 13 and Table 3 indicate that the average sensitivities and operation ranges of the proposed twisted dual-cycle fiber optic bending loss sensor head can be increased by increasing the number of steel wires and the radius of the steel wires, and by decreasing the distance between steel wires. Therefore, the sensitivities, sensing range, and the operating range of the proposed FOS head can be readily adjusted depending on the deformation characteristics of the measurement target.

## 5. Conclusions

A twisted dual-cycle bending structure for ingenerating optical fiber bending loss was proposed for strain measurement. The bending loss characteristics according to the distance between steel wires, wire radius, and number of steel wires were experimentally evaluated to verify the proposed method. The results showed that the twisted dual-cycle bending structure exhibits a linear relationship between detected optical power loss and the spring deflection length (z) corresponding to the applied force. These experimental results can be used for manufacturing an FRP intensity-based sensing element in an intensity-based FOS measurement system.

To apply the proposed FOS head to an experimental measurement, the twisted dual-cycle bending structure was bonded to the FRP, and tensile and three-point bending tests were conducted. Two FRP coupons were tested in each of seven cases and each coupon was tested five times. The measurement range, sensitivity, and average measurement errors of the tensile load and flexural strain were 4.5 kN and 1,760 *με*, 0.70 to 3.99 dB/kN and 0.930 to 6.554 dB/mm, and 57.7 N, and 42.6 *με*, respectively. The sensing range of FOS head were 82 to 138 mm according to configuration cases.

The advantage of the proposed FOS head structure is that the sensitivity and measurement area can be readily adjusted by changing the radius of the steel wires, the number of steel wires, and the distance between steel wires. The sensitivity can also be improved by reducing the distance between steel wires and/or increasing the radius of the steel wires. In addition, the measurement area of the proposed FOS head can be adjusted by changing the number of steel wires and the distance between steel wires. The bending loss characteristics depend on the FOS head steel wire radius, numbers, and distance. To determine the effects of these parameters, two samples in each of seven configuration cases of the proposed FOS head were bonded to FRP coupons and tensile and flexural strain tests were repeated five times. The manufactured sensor head with same configuration has similar characteristics of the sensitivity and the operating ranges. An additional advantage of FOS implemented with standard single-mode optical fibers are definite: An optical network can be used to obtain real-time measurement information. Many devices developed for optical communications like light source, optic circulator, optical coupler, fiber Bragg grating, wavelength division multiplexing can be used with FOS head for multi-point sensing. In addition, it can measure various types of measurands with arbitrary spatial distribution. We already proposed and demonstrated the self-referencing, intensity-based FOS [3]. The manufactured FOS head can be applied together with the self-referencing intensity-based fiber optic sensor interrogator presented in 2014 [3] that features functionality such as self-referencing, remote sensing, and multiple sensor heads with cascade and/or parallel forms. The proposed FOS head can be manufactured into a patch-type sensor that can be easily installed or removed, which enables more convenient and accurate measurement of structural behavior in the field.

## Figures and Tables

**Table 1:** Seven kinds of intensity-based FOS heads implemented with different conditions.

FOS Heads	$R_{wire}\;(\mathrm{mm})$	$N_{wire}$	$d_{wire}$ (mm)	Sensor Length (mm)	Insertion Loss (dB)	FOS Heads	$R_{wire}\;(\mathrm{mm})$	$N_{wire}$	$d_{wire}$ (mm)	Sensor Length (mm)	Insertion Loss (dB)
Case 1-1	1	6	14	110	2.43	Case 1-2	1	6	14	110	2.89
Case 2-1	0.75	6	14	110	1.17	Case 2-2	0.75	6	14	110	1.65
Case 3-1	1.5	6	14	110	9.93	Case 3-2	1.5	6	14	110	9.98
Case 4-1	1	4	14	82	2.43	Case 4-2	1	4	14	82	2.56
Case 5-1	1	8	14	138	2.80	Case 5-2	1	8	14	138	3.35
Case 6-1	1	6	12	100	5.24	Case 6-2	1	6	12	100	5.27
Case 7-1	1	6	16	120	1.42	Case 7-2	1	6	16	120	1.61

**Table 2:** Measurement results of tensile strain test.

FOS Heads	Average Sensitivity		Curve Fitting Results ($s\times F_{t}{}^{l})$				Error
	(dB/kN)	(dB/με)	s	l	R-Square	RMSE	Average (dB, N, με)
Case 1-1	1.33	0.00294	1.1530	1.101	0.9988	0.06000	0.046, 34.2, 15.5
Case 1-2	1.35	0.00299	0.9624	1.231	0.9995	0.04020	0.034, 24.9, 11.3
Case 2-1	0.70	0.00155	0.5698	1.161	0.9983	0.03962	0.032, 45.1, 20.4
Case 2-2	0.76	0.00168	0.4716	1.319	0.9992	0.02768	0.023, 30.1, 13.6
Case 3-1	3.99	0.00883	3.2550	1.140	0.9984	0.21370	0.164, 41.1, 18.6
Case 3-2	3.83	0.00847	3.0000	1.168	0.9976	0.24810	0.180, 46.9, 21.2
Case 4-1	1.00	0.00221	0.7599	1.191	0.9975	0.06675	0.042, 42.0, 19.0
Case 4-2	1.02	0.00226	0.7939	1.171	0.9996	0.02755	0.022, 21.9, 9.9
Case 5-1	2.40	0.00531	1.6120	1.269	0.9992	0.09092	0.077, 32.1, 14.5
Case 5-2	2.35	0.00520	2.0260	1.103	0.9996	0.06102	0.048, 20.4, 9.2
Case 6-1	2.58	0.00571	2.2290	1.102	0.9971	0.18360	0.149, 57.7, 26.1
Case 6-2	2.50	0.00553	2.2310	1.081	0.9976	0.16090	0.129, 51.8, 23.4
Case 7-1	1.05	0.00232	0.4587	1.556	0.9976	0.06938	0.060, 57.5, 26.0
Case 7-2	1.09	0.00241	0.4109	1.655	0.9973	0.07569	0.060, 55.1, 24.9

**Table 3:** Measurement results of three-point flexural test.

FOS Heads	Average Sensitivity		Curve Fitting Results ($h\times \varepsilon_{f}{}^{k})$				Error
	(dB/mm)	(dB/με)	h	k	R-Square	RMSE	Average (dB, με)
Case 1-1	1.758	0.002996	43.95	1.216	0.9958	0.10200	0.080, 26.59
Case 1-2	1.777	0.003029	49.14	1.276	0.9972	0.08437	0.070, 23.11
Case 2-1	0.938	0.001599	23.55	1.217	0.9973	0.04419	0.036, 22.62
Case 2-2	0.930	0.001585	29.78	1.362	0.9975	0.04135	0.032, 20.44
Case 3-1	6.267	0.010682	129.8	1.105	0.9981	0.24650	0.194, 18.17
Case 3-2	6.554	0.011172	131.1	1.070	0.9969	0.33200	0.256, 22.88
Case 4-1	1.377	0.002347	36.81	1.251	0.9902	0.12330	0.100, 42.58
Case 4-2	1.448	0.002469	36.80	1.227	0.9984	0.05245	0.042, 16.90
Case 5-1	2.976	0.005073	70.79	1.181	0.9983	0.10980	0.083, 16.30
Case 5-2	3.085	0.005258	66.04	1.129	0.9974	0.13950	0.112, 21.30
Case 6-1	3.294	0.005615	70.91	1.109	0.9964	0.18170	0.134, 23.92
Case 6-2	3.243	0.005529	69.4	1.131	0.9983	0.11960	0.097, 17.58
Case 7-1	1.118	0.001905	45.84	1.505	0.9981	0.04317	0.033, 17.09
Case 7-2	1.187	0.002024	50.45	1.529	0.9963	0.06420	0.050, 24.83
